# Maternal Ethanol Consumption Alters the Epigenotype and the Phenotype of Offspring in a Mouse Model

**DOI:** 10.1371/journal.pgen.1000811

**Published:** 2010-01-15

**Authors:** Nina Kaminen-Ahola, Arttu Ahola, Murat Maga, Kylie-Ann Mallitt, Paul Fahey, Timothy C. Cox, Emma Whitelaw, Suyinn Chong

**Affiliations:** 1 Division of Genetics and Population Health, Queensland Institute of Medical Research, Herston, Australia; 2 Department of Biological and Environmental Sciences, University of Helsinki, Helsinki, Finland; 3 Division of Craniofacial Medicine, Department of Pediatrics, University of Washington, Seattle, Washington, United States of America; 4 Griffith Medical Research College, Griffith University and the Queensland Institute of Medical Research, Herston, Australia; Massachusetts General Hospital, Howard Hughes Medical Institute, United States of America

## Abstract

Recent studies have shown that exposure to some nutritional supplements and chemicals *in utero* can affect the epigenome of the developing mouse embryo, resulting in adult disease. Our hypothesis is that epigenetics is also involved in the gestational programming of adult phenotype by alcohol. We have developed a model of gestational ethanol exposure in the mouse based on maternal *ad libitum* ingestion of 10% (v/v) ethanol between gestational days 0.5–8.5 and observed changes in the expression of an epigenetically-sensitive allele, *Agouti viable yellow* (*A^vy^*), in the offspring. We found that exposure to ethanol increases the probability of transcriptional silencing at this locus, resulting in more mice with an agouti-colored coat. As expected, transcriptional silencing correlated with hypermethylation at *A^vy^*. This demonstrates, for the first time, that ethanol can affect adult phenotype by altering the epigenotype of the early embryo. Interestingly, we also detected postnatal growth restriction and craniofacial dysmorphology reminiscent of fetal alcohol syndrome, in congenic *a/a* siblings of the *A^vy^* mice. These findings suggest that moderate ethanol exposure *in utero* is capable of inducing changes in the expression of genes other than *A^vy^*, a conclusion supported by our genome-wide analysis of gene expression in these mice. In addition, offspring of female mice given free access to 10% (v/v) ethanol for four days per week for ten weeks prior to conception also showed increased transcriptional silencing of the *A^vy^* allele. Our work raises the possibility of a role for epigenetics in the etiology of fetal alcohol spectrum disorders, and it provides a mouse model that will be a useful resource in the continued efforts to understand the consequences of gestational alcohol exposure at the molecular level.

## Author Summary

In humans it has been known for some time that exposure to environmental insults during pregnancy can harm a developing fetus and have life-long effects on the individual's health. A well known example is fetal alcohol syndrome, where the children of mothers that consume large amounts of alcohol during pregnancy exhibit growth retardation, changes to the shape and size of the skull, and central nervous system defects. At present the molecular events underlying fetal alcohol syndrome are unknown. We have developed a model of alcohol exposure in the mouse, in which the genetics and the environment can be strictly controlled. We find that chronic exposure of the fetus to a physiologically relevant amount of alcohol during the first half of pregnancy results in epigenetic changes at a sensitive reporter gene and produces fetal alcohol syndrome-like features in some mice. Our model is a useful tool to study the underlying causes of fetal alcohol syndrome, and our work raises the interesting possibility that the long-term physical effects of alcohol exposure during pregnancy are mediated by epigenetic changes established in the fetus and then faithfully remembered for a lifetime. In the future, such epigenetic changes could be used as markers for the preclinical diagnosis and treatment of fetal alcohol spectrum disorders.

## Introduction

While it is well-recognized that gestational exposure to environmental triggers can lead to compromised fetal development and adult disease in humans [1], the underlying molecular mechanisms remain unknown. There is increasing evidence in animal models that environmental factors can affect gene expression via epigenetic modifications such as DNA methylation [2]–[6]. One way of detecting such events is to use reporters whose expression is closely linked to their epigenetic state. Such epigenetically sensitive alleles are also known as metastable epialleles, and the best known example in the mouse is *Agouti viable yellow* (MGI:1855930) or *A^vy^* [7]. *A^vy^* is a dominant mutation of the murine *Agouti* (*A*) locus, caused by the insertion of an intracisternal A-particle (IAP) retrotransposon upstream of the *Agouti* coding exons. The activity of *A^vy^* is variable among genetically identical mice, resulting in mice with a range of coat colors; from yellow to mottled to agouti (termed pseudoagouti) [8]. The expression of *A^vy^* is known to correlate with DNA methylation at a cryptic long terminal repeat (LTR) promoter located at the 3′ end of the inserted IAP. Specifically, hypomethylation is associated with constitutive ectopic *Agouti* expression and a yellow coat, while hypermethylation correlates with cryptic promoter silencing and a pseudoagouti coat [9]. We have previously shown that DNA methylation at *A^vy^* is reprogrammed in early development at the same time that the rest of the genome is undergoing epigenetic reprogramming [10].

Alcohol consumption is widespread in our society, but it is also recognized as the leading preventable cause of birth defects and mental retardation [11],[12]. High levels of alcohol consumption during pregnancy can result in fetal alcohol syndrome (FAS) which is characterized by prenatal and postnatal growth restriction, craniofacial dysmorphology and structural abnormalities of the central nervous system. The clinical features of FAS are variable and include a range of other birth defects, as well as educational and behavioral problems [13]. This syndrome is the most extreme form of a range of disorders that are known as fetal alcohol spectrum disorders (FASDs) [14]. Approximately 5% of the children of mothers who have drunk heavily during pregnancy have FAS [15], and studies have shown that the dose, time and duration of ethanol exposure are critical [16],[17]. There are a number of mouse models of FAS that have reproduced some of the phenotypic characteristics of the human disorder, particularly the craniofacial abnormalities [16],[18],[19]. It should be noted that these studies used acute ethanol exposures between gestational days (GDs) 7 and 9 and high concentrations; generally two intraperitoneal injections of 0.015 ml of ∼25% (v/v) ethanol per gram of body weight over a 4 hour interval resulting in ataxia and lethargy. These studies only examined the fetal outcomes (GDs 8-18) of ethanol exposure and did not assay offspring either after birth or as adults. There are some rodent studies of the effects of gestational exposure to moderate amounts of ethanol, but these have only identified neurological and behavioral deficits [20].

The molecular mechanisms underlying FAS are unknown. Some studies have focused on the toxic effects of acetaldehyde, the first metabolite of ethanol [18],[21]. Acute ethanol exposure has also been found to result in increased cell death in the developing central nervous system and neurological anomalies in rodents and other animal models [22],[23]. The idea that epigenetic changes are involved has been raised but evidence in support of this hypothesis has, so far, been weak. Garro and colleagues [24] detected a small decrease in the level of global methylation of fetal DNA after acute ethanol administration from GDs 9-11. Bielawski *et al.* [25] reported decreased *DNA methyltransferase 1* (*Dnmt1*) messenger RNA levels in rat sperm after nine weeks of paternal ethanol exposure. Haycock and Ramsey [26] studied imprinting of the *H19/Igf2* in preimplantation mouse embryos after maternal ethanol exposure. Despite severe growth retardation of embryos, they did not find epigenetic changes at the *H19* imprinting control region.

Here we have developed a mouse model of chronic ethanol exposure (overt signs of intoxication are not observed) that produces measurable phenotypes in adults. We find that maternal ethanol consumption either before or after fertilization affects the expression of an epigenetically sensitive allele, *A^vy^*, in her offspring and that, at least in the latter case, can also impact postnatal body weight and skull size and shape in a manner consistent with FASD. Our work raises the possibility of a role for epigenetic reprogramming in the etiology of FASD and provides researchers with a relevant mouse model of the human disorder.

## Results

In this study, *A^vy^* was used primarily as a sensitive reporter of epigenetic changes in response to maternal ethanol consumption. The C57BL/6J mouse is null (*a*) at the *Agouti* locus, so it has a black coat color. *A^vy^* is a gain-of-function, semi-dominant mutation and so the coat color of heterozygous (*A^vy^/a*) mice in the C57BL/6J background is a direct read out of *A^vy^* transcriptional activity and DNA methylation. The nature of the matings used in this study, an *A^vy^/a* male crossed with an *a/a* female, means that only 50% of the offspring will inherit the *A^vy^* allele and be useful for coat color phenotyping. The remaining (*a/a*) offspring will be black. To study the effects of gestational ethanol exposure, female *a/a* C57BL/6J mice were supplied with 10% (v/v) ethanol in their drink bottles for eight days after fertilization by a congenic male carrying the *A^vy^* allele (n = 46 litters, 242 total offspring, 109 *A^vy^/a* offspring). To evaluate the effects of preconceptional ethanol exposure, female *a/a* mice were given 10% (v/v) ethanol for four days per week for ten weeks prior to fertilization (n = 22 litters, 131 total offspring, 69 *A^vy^/a* offspring). The *A^vy^* allele was passed through the male germ line to avoid the bias associated with maternal transmission, where epigenetic marks can be incompletely cleared between generations [9]. Control mice were given water instead of ethanol (n = 37 litters, 189 total offspring, 91 *A^vy^/a* offspring). Maternal ethanol exposure during gestation did not significantly alter Mendelian inheritance of the *A^vy^* allele (data not shown) or litter size (control 5.1±0.4, ethanol exposed 5.2±0.3, mean±SEM, Student's *t*-test, p = 0.9).

**Figure 1:**
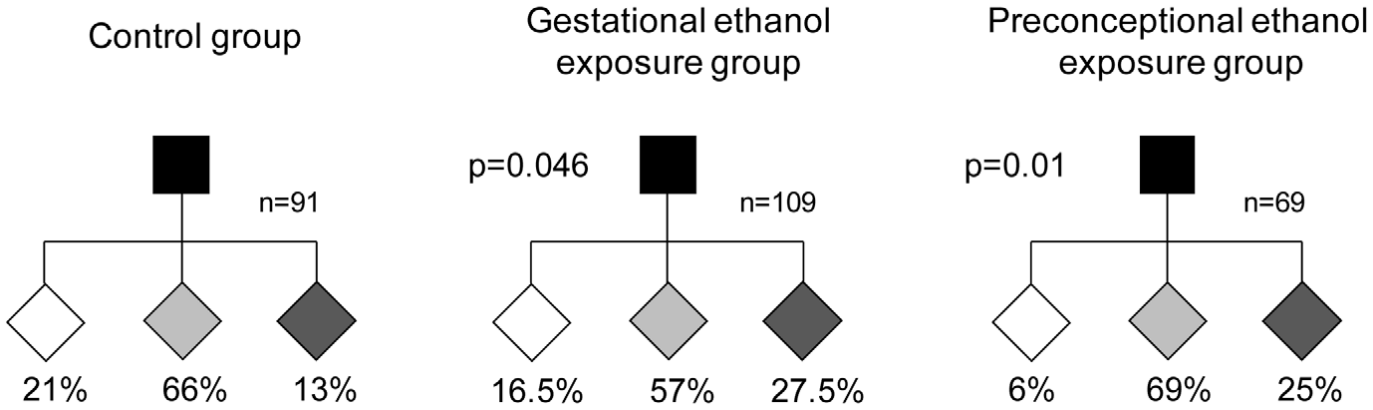
Gestational and preconceptional ethanol exposure produced a higher proportion of pseudoagouti *A^vy^* mice. In each pedigree, the black square represents the *a/a* dam and the diamonds represent her *A^vy^/a* offspring. *A^vy^/a* sires and all *a/a* offspring have been excluded from the pedigrees. White diamonds represent yellow offspring, light gray diamonds represent mottled offspring and dark gray diamonds represent pseudoagouti offspring. The percentage of offspring in each coat color category is indicated. The gestational ethanol exposure group and the preconceptional ethanol exposure group were statistically significantly different to the control group using Pearson's chi-square test (p<0.05).

**Figure 2:**
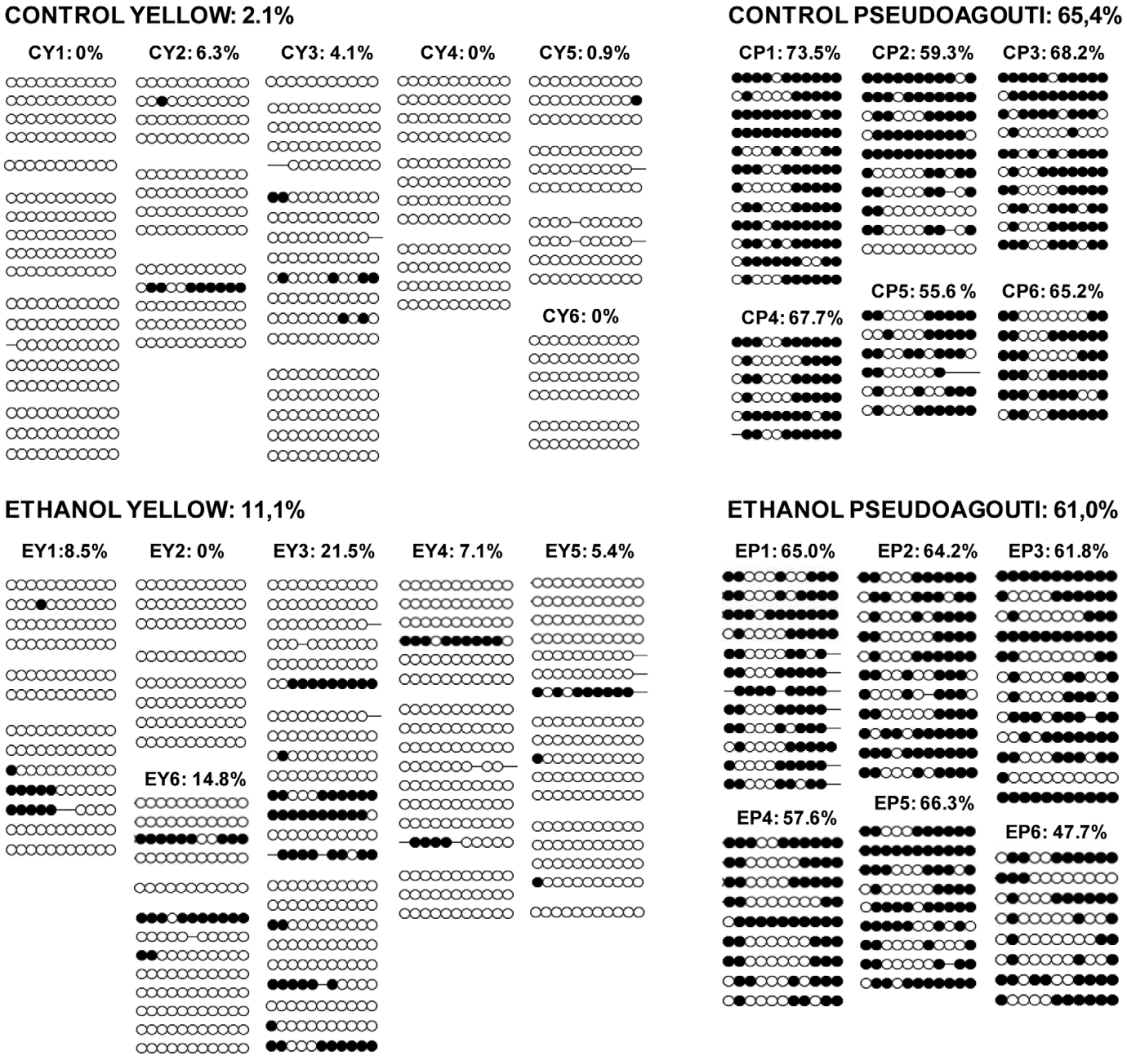
*A^vy^* methylation in control offspring and offspring exposed to ethanol *in utero*. Only mice with 100% yellow and 100% pseudoagouti coats were assayed. Control yellow (CY) offspring are numbered 1–6, control pseudoagouti (CP) offspring are numbered 1–6, ethanol yellow (EY) offspring are numbered 1–6 and ethanol pseudoagouti (EP) offspring are numbered 1–6. Methylation was analyzed by sequencing individual clones of PCR-amplified, bisulfite-converted tail genomic DNA. Each circle represents an individual CpG. Open circles indicate an unmethylated CpG, and closed circles represent a methylated CpG. Each line represents an individual clone and the methylation pattern of one allele in one cell. Each block of lines comprises clones derived from one bisulfite conversion. The total percentage of methylated CpGs is shown above each individual and group. There are more hypermethylated clones in yellow ethanol exposed mice (Pearson's chi-square tests. p<0.01). There is no effect of ethanol exposure on the methylation of *A^vy^* in pseudoagouti mice (Student's *t*-test, p = 0.27).

The establishment of epigenetic marks at *A^vy^* occurs during early embryogenesis and is a probabilistic event. The resulting variable expression of *A^vy^* among genetically identical mice produces individuals with a predictable range of coat colors. We found that, in the absence of any treatment, 21% of the offspring of *A^vy^/a* sires were yellow, 66% were mottled and 13% were pseudoagouti (Figure 1). Gestational ethanol exposure resulted in a higher proportion of pseudoagouti (Pearson's chi-square test, p<0.05). Twenty-eight percent of offspring were pseudoagouti compared with 13% in the control group (Figure 1). Preconceptional ethanol exposure produced a similar trend (Pearson's chi-square test, p<0.05). This shows that ethanol exposure can influence the establishment of *A^vy^* expression early in development. It increases the probability of transcriptional silencing at this particular locus. To confirm that the coat color correlated with DNA methylation at the *A^vy^* allele in gestationally exposed mice, 11 CpG dinucleotides in the LTR cryptic promoter of the *A^vy^* IAP were subjected to bisulfite sequencing (Figure 2). The results showed that, as expected, ethanol-exposed yellow mice were hypomethylated compared to ethanol-exposed pseudoagouti mice. Interestingly, atypical hypermethylated clones were found in five out of six yellow mice in the ethanol-exposed group, but they were clearly not sufficient to affect coat color. In the ethanol-exposed group 11% of the CpG dinucleotides were methylated compared to 2% in the control group. Using this measure a Student's *t*-test or non-parametric equivalent was unsuitable because the data did not meet the distribution requirements of being spread on a continuum. So we analyzed allele-specific methylation. In the ethanol-exposed group 23% of clones showed evidence of methylation, n = 91, compared with 8% of clones in the control group, n = 71 (Pearson's chi-square tests, p<0.01). In contrast, total DNA methylation level in ethanol exposed pseudoagouti mice (61%) was not significantly different to that observed in the controls (65%, Student's *t*-test, p = 0.27). Equivalent results were obtained from a random effects model which allowed for the clustering of clones within mice (p = 0.23). Bisulfite sequencing was also carried out on control and ethanol-exposed mottled mice and we found the results extremely variable from one mouse to the next within both groups (Figure S1). Presumably, this is the result of the small size of the tissue sample (tail tip). The variegated expression in mottled mice means that any one sample could represent only one clonal patch, which could harbour an active or an inactive *A^vy^* allele and not represent the true methylation state of the whole animal. For this reason mottled mice were not used in our analyses.

The effects of *A^vy^* expression are pleiotropic. For example, yellow mice exhibit hyperphagia, hyperglycemia, non-insulin-dependent diabetes and adult onset obesity [27]. We did not assay these other phenotypes following ethanol exposure in our mice as their relevance to humans is questionable since no human ortholog of the *A^vy^* allele has been identified. So, while *A^vy^* was initially useful as a sensitive indicator of epigenetic changes, any further study of FAS-like phenotypes must necessarily focus elsewhere in the genome. For this reason and the fact that variable *Agouti* expression would confound many phenotypes, all subsequent analyses were performed on the congenic *a/a* siblings of the *A^vy^* mice. In the mouse, IAPs are present at approximately 1,000 copies per haploid genome [28]. To see if gestational ethanol exposure changed the methylation level of IAPs globally, we performed bisulfite sequencing using PCR primers that anneal to all IAP LTRs and analyzed ten CpG sites in both the tail and forebrains of *a/a* mice. Tail DNA from eight ethanol-exposed mice (66 clones total) and eight control mice (65 clones total) were compared, and forebrain DNA from five ethanol-exposed mice (33 clones total) and five control mice (43 clones total) were compared using the Student's *t*-test. All samples were highly methylated and no differences between the ethanol exposure group and controls were detected (Figure S2). This suggests that only a subset of IAPs, perhaps those that are usually hypomethylated, are sensitive to ethanol exposure.

To detect changes in gene expression genome-wide, we performed expression arrays with liver tissue. The benefit of using liver is its homogeneity; it consists mainly of hepatocytes and consequently subtle changes will be detectable. We compared gene expression between age-matched male mice from the gestational ethanol group (three samples) and controls (four samples). In addition to inter-individual variation, some of the genes were consistently differentially expressed in the ethanol-exposed group (Table S1). Twelve genes were significantly down-regulated (p<0.05) in the ethanol-exposed mice. Three of these; *LIM domain and actin binding 1* (*Lima1*), also known as *Eplin*, *Suppressor of cytokine signaling 2* (*Socs2*) and *CDK5 and Abl enzyme substrate 1(Cables1)* have been associated with growth [29]–[32]. Three; *Socs2*, *Very low density lipoprotein receptor* (*Vldlr*) and *Cables1* have been associated with development of the nervous system [33]–[37] and one, *Hepcidin antimicrobial peptide* (*Hamp1*), has been reported to be down-regulated in the livers of alcohol-fed rats [38].

**Figure 3:**
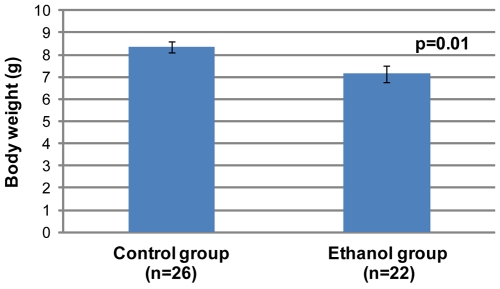
Offspring in the gestational ethanol exposure group have a statistically significantly lower mean weight than the control group (Student's *t*-test, p<0.05). All were *a/a* pups from first litters. Pups were weighed at 3 weeks of age and came from litter sizes of 4 and 5. The graph shows mean±SEM.

We next focused on identifying the characteristic features of FAS in *a/a* pups exposed to moderate levels of alcohol *in utero*. All pups (from first litters) were weighed at three weeks of age. It was particularly important not to study *A^vy^* mice in these experiments because of the effects on body weight due to *Agouti* expression. Because litter size is known to influence body weight at weaning, we initially restricted our analysis to litters of 4–5 pups. The gestational ethanol exposure group consisted of 22 offspring, while the control group consisted of 26 offspring. The results (Figure 3) show that the mean weight of offspring of dams that consumed ethanol were significantly lower than that of controls (Student's *t*-test, p<0.05). A second analysis included litter size as a random effect. Analysis of Variance of weight at 3 weeks, after adjustment for litter size, confirmed that the mean weight of the ethanol exposed group (n = 73) was statistically significantly smaller than the mean weight of the controls (n = 44, p<0.001, data not shown).

**Figure 4:**
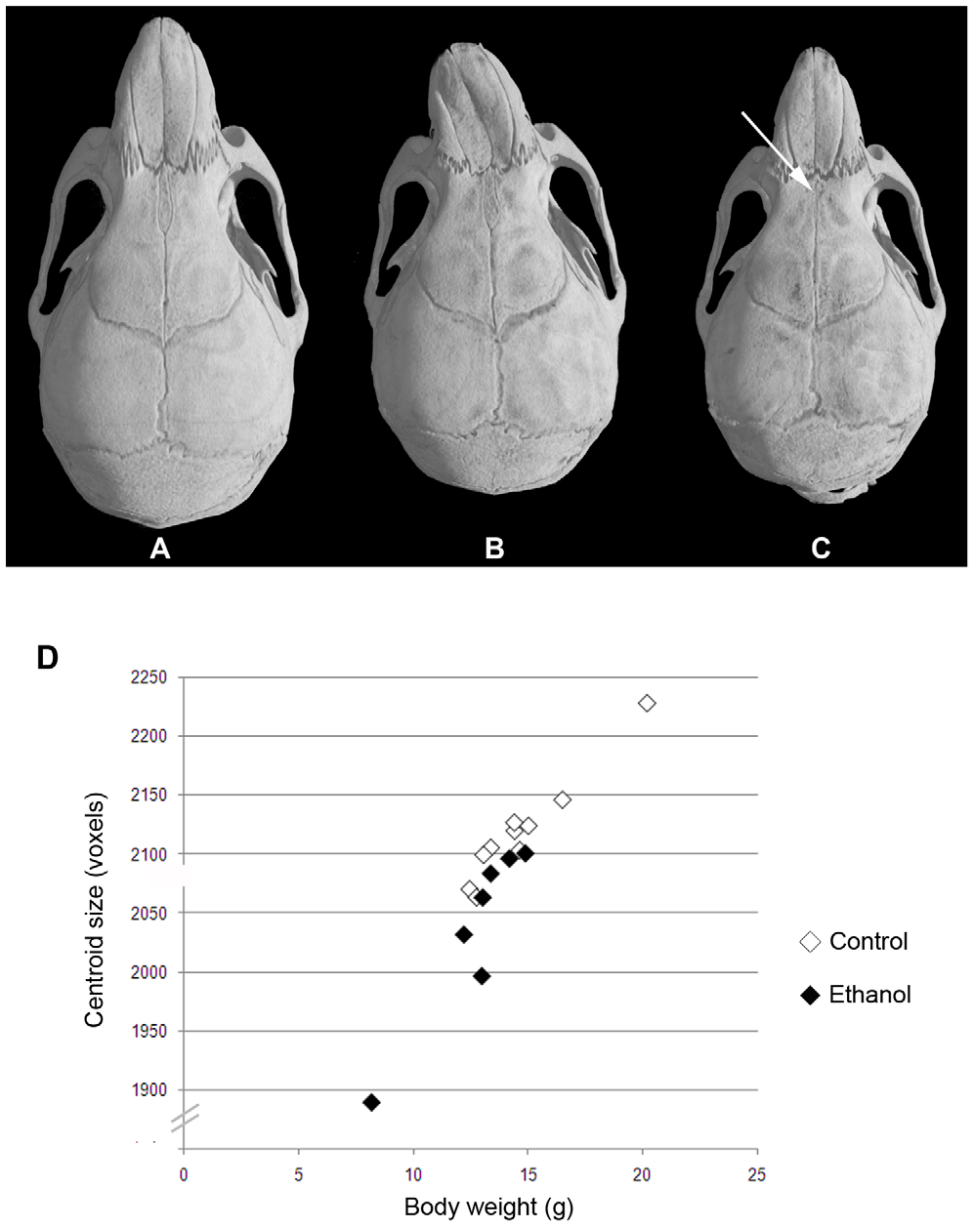
Variable midfacial dysmorphism and microcephaly in *a/a* offspring of mothers that consumed ethanol during gestation. The top panel shows 3D reconstructions of skull microCT data from 28–30 day old mice. (A) Control female. (B) Ethanol-exposed male showing marked leftward deviation of the midface. (C) Ethanol-exposed female showing an almost absent interfrontal bone that is normally characteristic of untreated C57BL6/J mice. (D) Graph of centroid size (used as an estimate of overall cranial size) against body weight for control (n = 10) and ethanol exposed (n = 7) mice at 28–30 days old. Centroid size was determined by summing the distances from each landmark to the centroid for each individual. Centroid size is highly correlated with body weight in both treatment groups, but those in the ethanol group are on average smaller than those in the controls (ANOVA, p<0.05).

The heads of 28–30 day old *a/a* mice (seven mice from gestational ethanol exposure group and 10 control mice) were subjected to micro-computed tomography, and three-dimensional computer-reconstructions at 18 µm resolution were made of each skull. Visual inspection of the reconstructions revealed an obviously smaller skull size in the ethanol group compared to controls. In addition, differences in shape in a few, but not all, individuals in the ethanol group were apparent. Most notable was the marked leftward deviation of the midface in one male (Figure 4B) and a significantly reduced interfrontal bone in one female (Figure 4C). To provide more quantitative information on skull shape, the 3D co-ordinates of thirty-four landmarks were recorded for each skull and used in various mathematical-based shape and form analyses.

There are two classic approaches in geometric morphometrics: superimposition based methods such as Generalized Procrustes Analysis (GPA) [39]–[42] or invariant analyses of shape, such as Euclidean Distance Matrix Analysis (EDMA) [43],[44]. GPA involves translation, rotation and scaling of landmark data through an iterative process during which the distances between the shapes are minimized by applying least-squares criteria. We used GPA to test for the mean shape difference between the groups and to quantify and visualize localized differences in the cranial shape. We also applied the multivariate ordination method Canonical Variates Analyses to the output of the GPA. EDMA, in contrast, uses a coordinate-free (or invariant) approach in which all the landmarks are converted into a matrix of inter-landmark distances [44],[45]. We used EDMA to find the landmark pairs that show the most difference between two groups.

**Figure 5:**
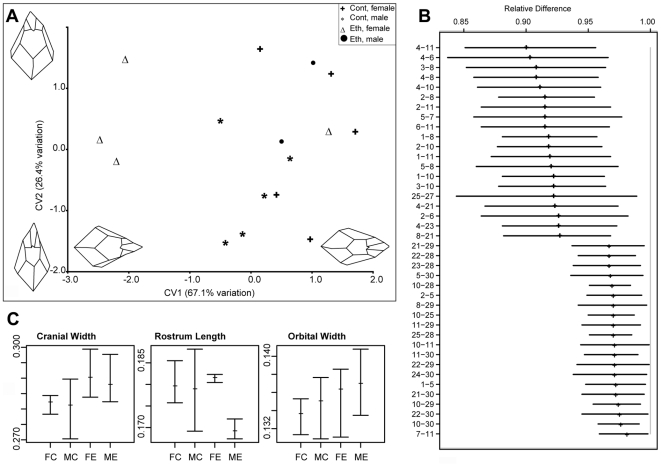
Quantitative analysis of the effects of gestational exposure to ethanol on skull shape. (A) Output of the CVA on the procrustes coordinates. Two components account for more than 90% of the total variation. CV1 clearly discriminates ethanol treated female specimens from the rest of the samples. Both male and female ethanol specimens have CV2 values close to (and above) 0, whereas controls span the entire range. Changes in the skull morphology were visualized by using the loadings of the first two canonical variates. Negative values of CV1 indicate a relatively wider skull and relatively longer rostrum. Positive values of CV2 indicates relatively wider orbits (as measured by landmarks 7–11), and shorter rostrum. (B) Output of the EDMA form difference matrix. Out of the possible 561 interlandmark distances, the top and bottom 20 are shown. Values are reported as the proportion of ethanols to controls for each interlandmark distance. None of the confidence intervals for the reported distances crosses 1.0, indicating that all the dimensions are significantly lower in the ethanol specimens. The higher values at the bottom of the graph indicate relatively expanded dimensions for ethanols. 90% confidence intervals were estimated by the non-parametric bootstrap method. For landmark descriptions and positions, see Text S1 and Figure S3. (C) Mean relative size of selected linear dimensions in ethanol and control groups. Tails indicate the observed range. Measurements are divided by the centroid size to remove the size effect. Cranial width is measured as the distance between landmarks 15 and 17; rostrum length is measured as the distance between landmark 9 and center of landmarks 1 and 2; orbital width is measured by the distance between landmark 7 and 11. Abbreviations: FC, female control; MC, male control; FE, female ethanol; ME, male ethanol. Goodall's F test was used to test for statistical significance of mean shape differences among groups.

Analysis of skull centroid sizes confirmed the observations of statistically significantly reduced cranial size in the ethanol group, even when the smaller body weight is taken into account (ANOVA, p<0.05; Figure 4D). Although the severe leftward deviation of the one male skull is biologically highly relevant, we chose to exclude this sample from subsequent shape analyses because of its significant impact on the results. This permitted us to assess the significance of other more subtle changes. However, it was included in the univariate analyses of relative cranial dimensions. In the absence of this outlier, CVA still revealed greater variation in overall craniofacial shape within the ethanol group (Figure 5A). Canonical variate 1 (CV1) clearly separated the females in the ethanol group from other skulls, suggesting a more pronounced effect on female skull shape. Notably, all the females as well as the included males from the ethanol group had positive values for CV2, whereas the controls spanned both negative and positive values (see Figure 5A), indicative of a similar trend in shape alteration in response to this level of gestational exposure to ethanol. One female from the ethanol group appeared to be unaffected in terms of craniofacial shape and grouped with the control females in all analyses.

We then used EDMA to assess the differences in form between the ethanol and control groups. Analysis of the 561 possible inter-landmark measurement combinations assessed by the 34 assigned landmarks (i.e. 34(34-1)/2) demonstrate that the majority show a consistent ratio below one, indicative of the fact that they are changed only relative to skull size and do not reflect localized altered shape. Nevertheless, numerous inter-landmark measures were shown to be significantly different from this mean form. The twenty most significant differences (α = 0.1) in either direction from the mean form are shown in Figure 5B. Strikingly, almost all of these forty most significant differences pertain to midfacial and palatal inter-landmark measures, highlighting the sensitivity of this region to the ethanol. In particular, these data reveal that the ethanol group as a whole have a relatively wider inter-orbital distance (inter-landmark measure 7–11), yet relatively shorter midface than controls (reflected in multiple inter-landmark measures). This is consistent with the CVA findings.

A univariate analysis of inter-landmark distances (normalized to centroid size) also supported these differences between the ethanol and control groups, in particular, confirming the greater relative cranial and inter-orbital width in both males and females compared to the sex-matched controls (Figure 5C). Females from the ethanol group also showed greater variation in ‘nare’ height (data not shown), while males from the ethanol group showed reduced rostrum length (Figure 5C). Although less severe than the changes found with acute ethanol exposure in the mouse [16], many of these differences are reminiscent of the facial changes seen in individuals presenting the milder end of fetal alcohol spectrum disorder, and support the notion that this ethanol regime provides a useful and relevant model for the effects of ethanol intake in humans.

## Discussion

The *A^vy^* allele has been called an epigenetic biosensor for environmental effects on the fetus [46]. Previous studies with this allele have identified a number of nutritional factors or toxic agents that affect expression and epigenetic regulation of *A^vy^* in offspring exposed *in utero*. For example, the addition of methyl supplements or an isoflavone (called genistein) to the diet causes hypermethylation of *A^vy^* and a shift to a pseudoagouti coat color [3],[4],[47], whereas bisphenol A, a chemical used in the manufacture of polycarbonate plastics causes a shift towards hypomethylation and a yellow coat [48]. We have used the *A^vy^* allele to investigate the epigenetic effects of exposure to ethanol, and established two models of moderate exposure in the mouse. The first involves exposure during the first eight days after fertilization; a period that encompasses pre-implantation, implantation and the first two days of gastrulation. This model simulates the effects of ethanol exposure during the first trimester of pregnancy in humans. Based on previous studies [49] we estimated that the peak blood alcohol level in our model is approximately 0.12%, which is a realistic human exposure. A World Health Organization (WHO) report shows that the maximum legal blood alcohol level for driving in Organization for Economic Cooperation and Development (OECD) countries varies from 0.02–0.08% [50]. The second model involves ethanol consumption for ten weeks immediately prior to fertilization; a period that encompasses multiple cycles of oocyte maturation and ovulation. In mammals, oocyte maturation is characterized by the resumption of meiosis, extrusion of the first polar body and the accumulation of RNAs and proteins in the cytoplasm in preparation for fertilization. Studies of epigenetic reprogramming have shown that following global DNA demethylation, the period of genome-wide remethylation coincides with implantation (for embryos) and oocyte growth (for female germ cells) [51].

Our results demonstrate that gestational ethanol exposure increases the likelihood of transcriptional silencing at *A^vy^*, resulting in an agouti-colored coat. It is worth emphasizing that despite being genetically identical, not all *A^vy^* mice become pseudoagouti; rather there is a subtle ∼15% increase in the proportion of pseudoagouti offspring. Previous studies have demonstrated that there is a tight correlation between DNA methylation at *A^vy^* and coat color [9]. As expected, bisulfite sequencing showed that the observed coat colors correlated with DNA methylation status in all cases. We did observe atypical hypermethylated clones in five of six yellow mice from the ethanol exposed group that, while not sufficient to change coat color, may reflect a tendency towards increased DNA methylation in this group. Preconceptional ethanol exposure produced a similar shift towards pseudoagouti in *A^vy^* offspring. It is likely that the two types of ethanol exposure have different modes of action on *A^vy^* because this allele is paternally-derived and not present in unfertilized oocytes. Consequently, the effects of preconceptional ethanol exposure on *A^vy^* expression will be indirect, and further work will be required to understand this mechanism. It is of interest that the coat color changes observed in *A^vy^* mice exposed to a methyl rich diet can be inherited across generations [52]. It is therefore possible that the altered coat color following alcohol exposure could also be transmitted to the next generation, but was beyond the scope of this study.

Our model of moderate gestational ethanol exposure produces a postnatal growth restriction phenotype and craniofacial dysmorphism in line with those seen with FASD in humans. It is possible that the postnatal growth restriction phenotype is an indirect effect; for example, the offspring may be smaller because of deficient maternal care between birth and weaning. It is unlikely that the dam would have been intoxicated or even experiencing ethanol withdrawal symptoms in the postpartum period since ethanol exposure ceased at GD 8.5 and water was consumed for the rest of gestation (∼11.5 days) and throughout nursing (21 days). Regardless of whether the phenotype is a direct physiological consequence of exposure *in utero* or the indirect result of altered maternal behavior, we would argue that it is ultimately a product of exposure to ethanol. Interestingly the effects on skull shape in these mice, like the coat color presentation in *A^vy^* mice, are variable despite the fact that the mice are isogenic. Marked variability in phenotype has also been recognized in humans in which not all children of heavily drinking mothers have the typical FAS facial phenotype; the others falling in the continuum of FASDs [14]. While this variability has been attributed to genetic differences, and differences in the level/timing of exposure, it may also be a consequence of stochastic establishment of epigenetic state.

The mechanism by which ethanol alters the establishment of epigenetic state at *A^vy^* is not known. It has been shown that chronic ethanol consumption can alter DNA methylation by changing the levels of S-adenosylmethionine (SAM), which donates methyl groups to cytosine [53],[54]. It is also known that chronic or acute ethanol consumption can cause post-translational histone modifications in rat tissues [55]–[59]. The effect of ethanol on the developing embryo has been less studied at the molecular level. Candidate gene and microarray analyses have detected changes in the level of expression (both up- and down-regulation) of numerous genes [60]–[62] and decreased global DNA methylation in midgestation embryos has been reported following acute ethanol treatment [24]. Recent studies have also reported altered regulation of several microRNAs by ethanol suggesting a possible role for these RNA species in fetal alcohol syndrome [63],[64].

Our gestational exposure experiments demonstrate that the epigenome is vulnerable to ethanol during early embryogenesis, a time when the DNA synthetic rate is high and there is genome-wide epigenetic reprogramming. Our preconceptional ethanol exposure experiments show that changes in the maturing oocyte (another period in development when there are widespread changes to the epigenome) can also affect offspring phenotype. The identification of microcephaly and midfacial dysmorphism in our gestational exposure model suggests effects on genes other than *A^vy^*. Our preliminary genome-wide gene expression analyses of liver in ethanol exposed mice revealed twelve consistently down-regulated and three up-regulated genes. Ongoing work will determine if the expression of these same genes has been changed in other tissues and whether it correlates with alterations in methylation level. The variable and subtle nature of the observed phenotypes will make this work challenging, but our ultimate goal is to gain a better understanding of the molecular processes underlying FASDs.

## Materials and Methods

### Ethics Statement

All animals were handled in strict accordance with good animal practice as defined by the relevant national and/or local animal welfare bodies, and all animal work was approved by the Animal Ethics Committee of the Queensland Institute of Medical Research (P986, A0606-609M).

### Study Design and Animals

The mice used in this study were inbred, genetically identical, C57BL/6J and all environmental factors (e.g. cage type, environmental enrichment) were standardized. We chose a voluntary consumption strategy for ethanol exposure instead of intraperitoneal injections or intragastric administration because it produces the least amount of maternal stress. C57BL/6 mice are also known to have a strong drinking preference for 10% (v/v) ethanol over water making them ideal for the study [65],[66].

For gestational ethanol exposure, single mottled *A^vy^/a* males were caged with single 6–14 week old *a/a* females. The majority of females were 6–8 weeks old and virgins in both ethanol-exposured and control groups. The females were checked each morning for a vaginal plug which indicated that mating had taken place. The day of plugging was designated GD 0.5, the male was removed from the cage and the water bottle was replaced with one containing 10% (v/v) ethanol. Pregnant females were allowed free access to the drink bottle and food at all times. The ethanol solution was changed and consumption (ml) was measured every 24 hours. The average daily consumption was 3.1±0.4 ml of 10% (v/v) ethanol (or 12 g ethanol/kg body weight/day) which was not statistically significantly different from the average daily water consumption of control mice (Student's *t*-test, two tailed, p = 0.8). It has been shown that in female mice, voluntary consumption of 10% (v/v) ethanol at 14 g ethanol/kg body weight/day produces an average peak blood alcohol level of ∼120 mg/dl [49]. Only one out of 47 females tested refused to drink ethanol in the initial 24 hour period and was excluded from the analysis. On the final day of exposure, GD 8.5, the ethanol bottle was replaced with a bottle containing water. All dams were subjected to only one cycle of ethanol exposure. Offspring were left with their mothers until weaning (at 3 weeks of age), when their coat color was recorded (*A^vy^/a* mice) or they were weighed or subjected to micro-computed tomography (*a/a* mice). For preconceptional ethanol exposure, 6 week old *a/a* female mice were given 10% (v/v) ethanol for 4 days per week (4 days ethanol followed by 3 days water) for ten weeks. After treatment, at 18–22 weeks age, they were mated with mottled *A^vy^/a* males. *A^vy^/a* offspring were weaned and phenotyped for coat color at three weeks of age. All ethanol and control exposures were performed in parallel so that exactly the same animal house conditions were experienced for all experiments.

The coat color of *A^vy^/a* offspring was visually classified by a single trained observer (NK-A) and placed into one of five categories: yellow (>95% yellow), yellow/mottled (75–95% yellow), mottled (25–74% yellow or 25–74% agouti), pseudoagouti/mottled (75–95% agouti) or pseudoagouti (>95% agouti). In the final analysis these categories were combined into three classes: yellow, mottled (comprised of yellow/mottled, mottled and pseudoagouti/mottled) and pseudoagouti.

### Bisulfite Sequencing

For bisulfite sequencing of the *A^vy^* allele, 200–400 ng of tail genomic DNA was embedded in agarose and then treated with sodium bisulfite as described previously [10]. The bisulfite-treated DNA was resuspended in 30 µl of water and 5 µl was used in the primary PCR followed by a semi-nested PCR with 2–5 µl of template (primers were forward 5′ gaaaagagagtaagaagtaagagagagag 3′, reverse 5′ aaaatttaacacataccttctaaaaccccc 3′ and semi-nested reverse 5′ actccctcttctaaaactacaaaaactc 3′) [10]. One bisulfite conversion and PCR was performed for each pseudoagouti sample, while 3–5 independent conversions and 3 PCRs/conversion were performed for each yellow sample. Global IAP LTR sequences were amplified from bisulfite-converted tail and forebrain DNA using universal IAP primers; forward 5′ ttgatagttgtgttttaagtggtaaataaa 3′ and reverse 5′ aaaacaccacaaaccaaaatcttctac 3′ [67]. An agarose-only (no template) control was always included and the experiment was only continued if the agarose control was negative at the end of the semi-nested PCR. PCR fragments were gel-isolated and subcloned into the pGEM-T vector (Promega, Madison, Wisconsin, United States). Individually sequenced clones were analyzed with BiQ Analyzer [68]. To avoid bias, clones from the same PCR were only accepted if they differed by either CpG or non-CpG methylation. Any clones with lower than 90% conversion rate were also excluded from the dataset.

### Gene Expression Arrays

To detect possible changes in gene expression in gestational ethanol exposure mice compared to the controls, we used the MouseWG-6 v2.0 Expression BeadChips (Illumina). We extracted total liver RNA from 28 days old males from control and gestational ethanol groups, using a Qiagen RNeasy Plus-kit (Qiagen). We used a Bioanalyzer (Agilent RNA 6000 Nano, Agilent) to confirm the quality of RNA and accepted only samples with RNA Integrity Numbers (RINs) above 9. We amplified RNA using an Illumina TotalPrep RNA Amplification Kit and performed a Whole-Genome Gene Expression Direct Hybridization Assay (Illumina). The gene expression data from scanned microarray images generated by the Illumina BeadArrayTM Reader was analysed by the GenomeStudio Gene Expression Module (Illumina) by using probe information. Four control samples from two litters and three gestational ethanol exposure samples from three litters were analysed.

### Analysis of Skull Morphology

Seventeen *a/a* mice (ten controls and seven ethanol exposed mice) aged between 28 and 30 days were subjected to micro-computed tomography using a SkyScan 1076 microtomograph at the Small Animal Tomographic Analysis Facility located at the University of Washington. The sex and treatment breakdown of the microCT samples is female ethanol (n = 4), female control (n = 5), male ethanol (n = 3) and male control (n = 5). Specimens were scanned at 18 micron resolution (65 kV, 150 mA, 1.0 mm Al filter) and reconstructed as series of 8-bit grayscale images. Three-dimensional models of the skulls were generated using the thresholding algorithm in Analyze 3D (Mayo Clinic, version 9.0). A grayscale value of 55 was determined to be the optimum threshold value to remove the soft tissue structures and scan noise while keeping the skull morphology intact, and was used for all specimens. Using the point measurement tool of Analyze, 35 landmarks were collected from each specimen (Text S1 and Figure S3). Specimens were digitized by the same observer (MM) to reduce inter-observer error. Visualizations showed that landmark 31 could not be accurately determined in every specimen because of the occasional fusion of the presphenoid and basisphenoid bones. Because geometric morphometrics requires homologous landmarks collected from every specimen, this landmark was omitted in subsequent analyses.

Landmark data were fed into various morphometric packages. Using the R statistical package [69], linear measurements of certain common cranial dimensions were calculated from the landmark coordinates and normalized to their respective skull centroid sizes. Generalized Procrustes Analysis (GPA) was also conducted in R by using the SHAPES module. Goodall's F test was used to test for statistical significance of mean shape differences among groups. The Canonical Variates Analysis (CVA) was conducted in the MorphoJ package [70]. The loadings of the canonical variates 1 and 2 were used to visualize the cranial shape changes depicted by each axis.

The WinEDMA package [71] was used to conduct Euclidean Distance Matrix Analysis. We used the FORM procedure of WinEDMA to find the landmark pairs that significantly differed between two mean forms (i.e., ethanols and controls) as measured by the form difference matrix. Following Lele and Richtsmeier [45], the 90% confidence intervals for the pairwise ratios were calculated by bootstrapping the form difference matrix 1000 times.

## Supporting Information

Figure S1 *A^vy^* methylation in control offspring and offspring exposed to ethanol *in utero* in mottled mice. Only mice with 50% yellow/50% pseudoagouti coats were assayed.
(1.01 MB TIF)

Figure S2 Global IAP methylation in control offspring and in offspring exposed to ethanol *in utero*. Methylation was analyzed by sequencing individual clones of PCR-amplified, bisulfite-converted forebrain and tail genomic DNA.
(0.98 MB TIF)

Figure S3 Landmark positions.
(9.62 MB TIF)

Table S1 Summary of significantly up- and down-regulated genes in liver following ethanol exposure *in utero*. The Diff Score is a transformation of the p-value that provides directionality to the p-value based on the difference between the average signal in the control group versus the ethanol exposed group. For p-values of 0.05, 0.01 and 0.001 the Diff Scores are ±13, ±20, and ±30, respectively.
(0.03 MB XLS)

Text S1 Landmark descriptions.
(0.03 MB DOC)
