# Supplementary figures and images for: Maternal Ethanol Consumption Alters the Epigenotype and the Phenotype of Offspring in a Mouse Model
Source: PLoS Genet. 2010 Jan 15;6(1):e1000811. doi: 10.1371/journal.pgen.1000811 (PMC2797299; doi:10.1371/journal.pgen.1000811)

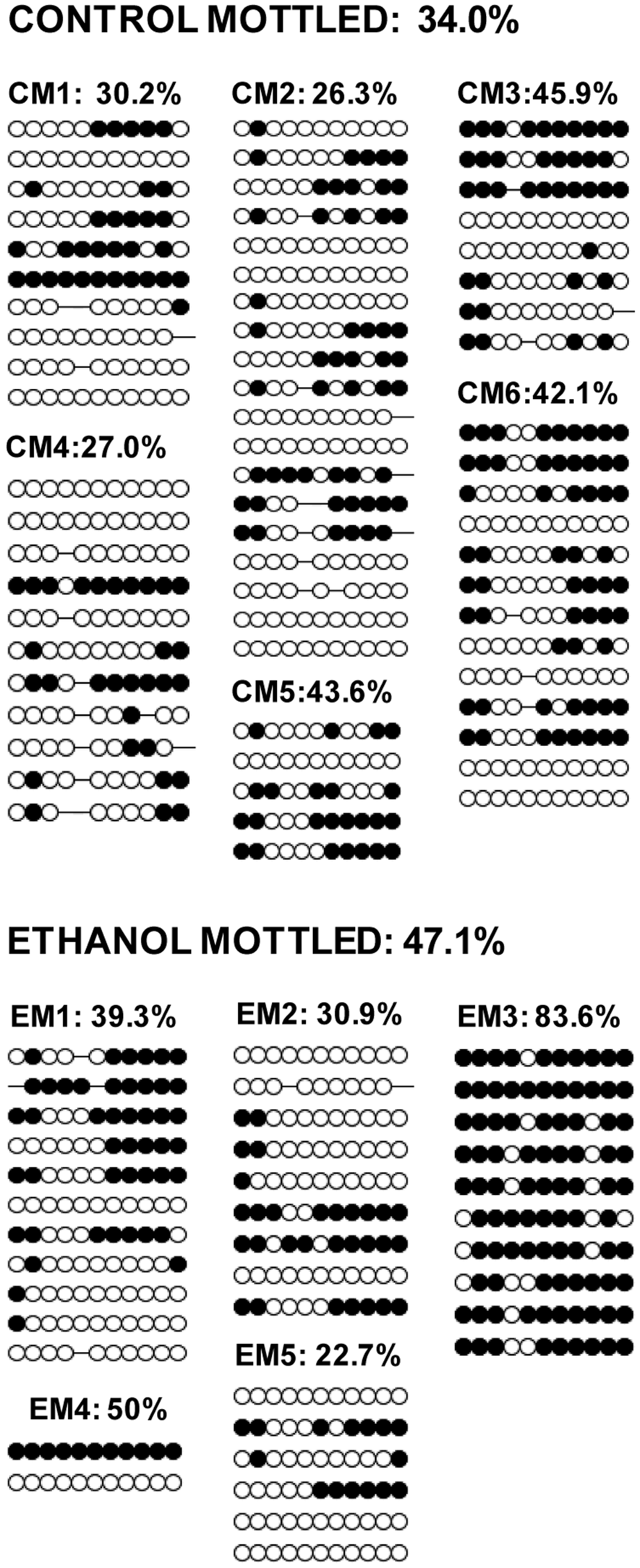

Supplement: Figure S1 — Avy methylation in control offspring and offspring exposed to ethanol in utero in mottled mice. Only mice with 50% yellow/50% pseudoagouti coats were assayed. (1.01 MB TIF) [file pgen.1000811.s001.tif]

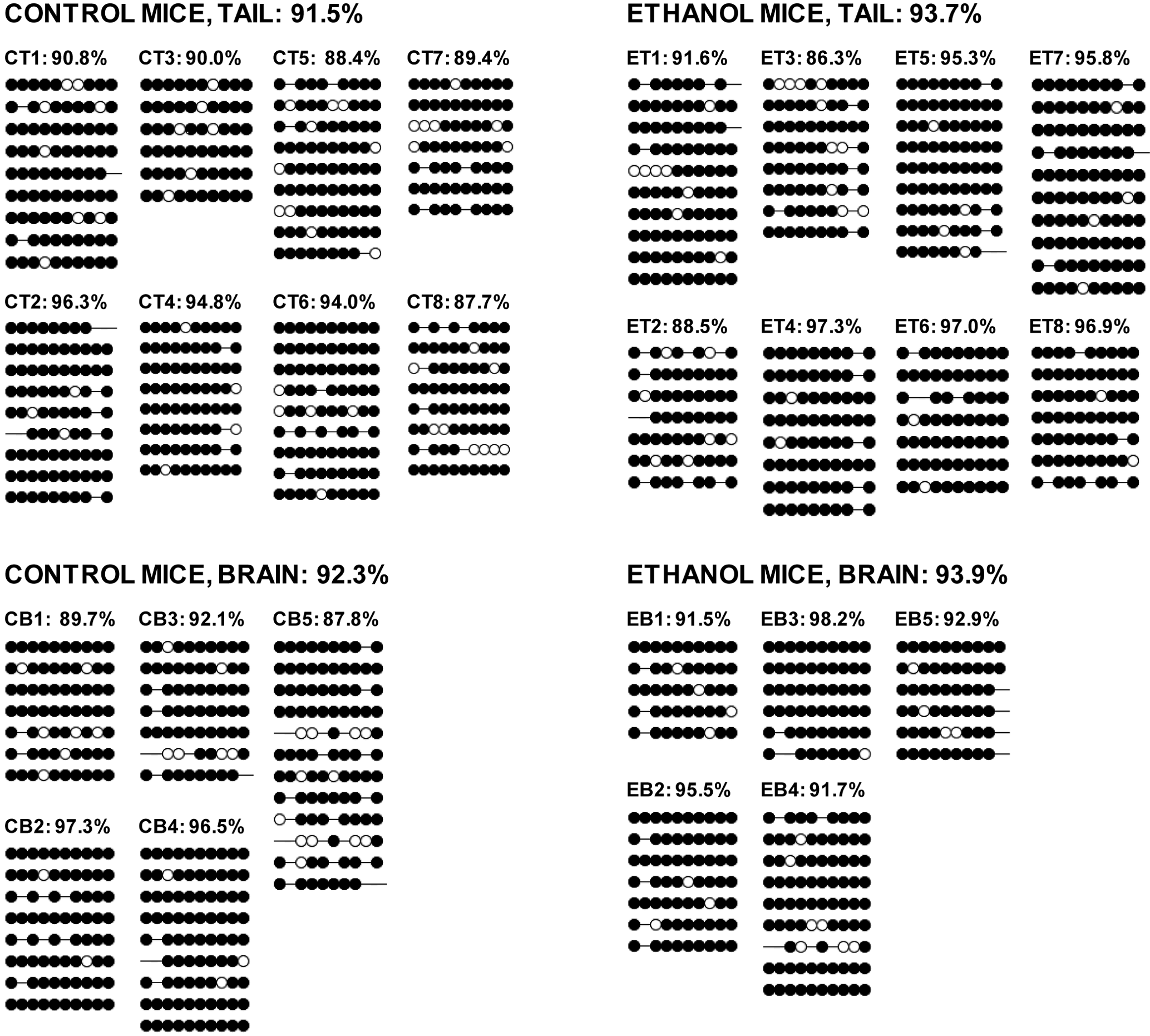

Supplement: Figure S2 — Global IAP methylation in control offspring and in offspring exposed to ethanol in utero. Methylation was analyzed by sequencing individual clones of PCR-amplified, bisulfite-converted forebrain and tail genomic DNA. (0.98 MB TIF) [file pgen.1000811.s002.tif]

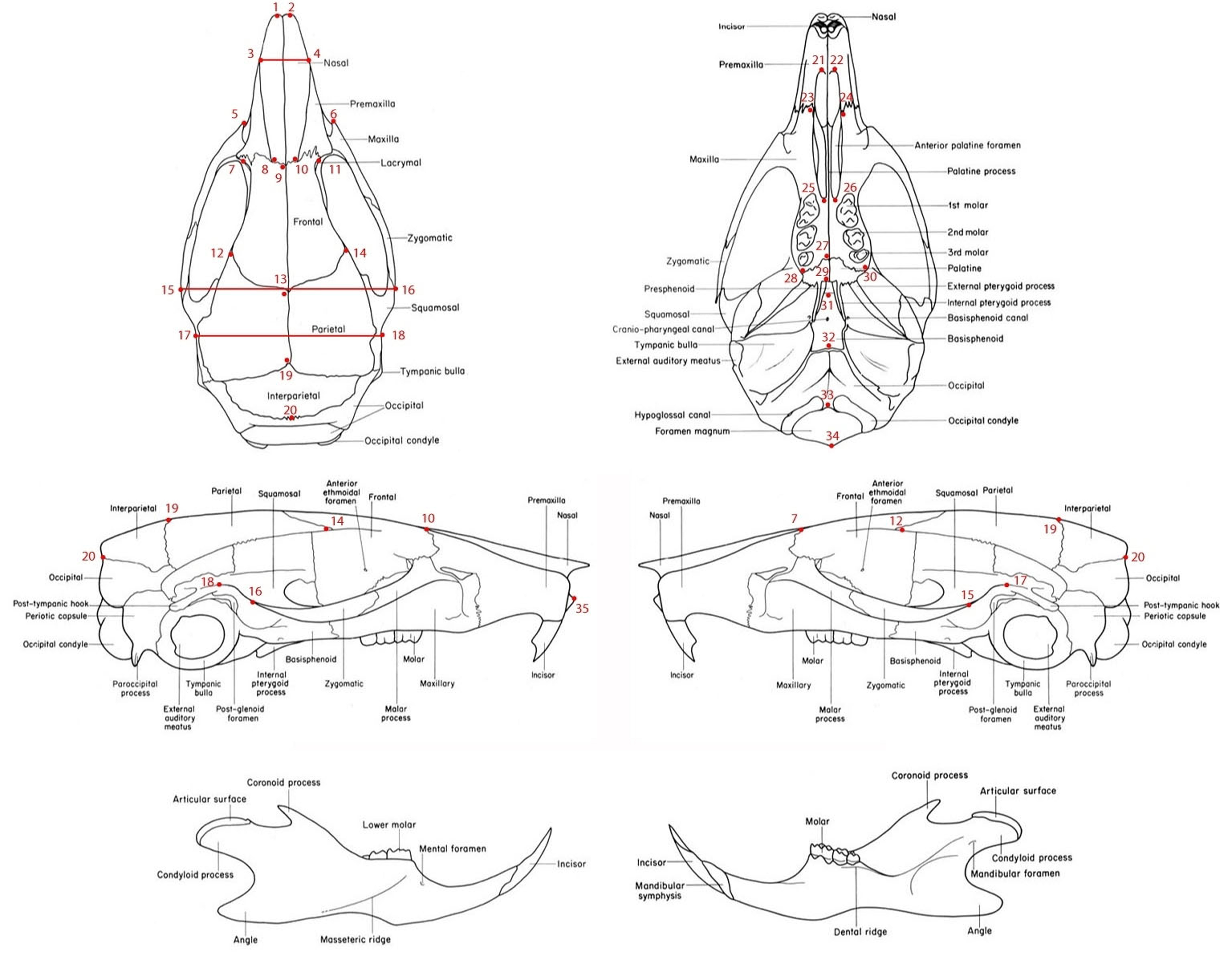

Supplement: Figure S3 — Landmark positions. (9.62 MB TIF) [file pgen.1000811.s003.tif]
