# Supplementary material for: Maternal Ethanol Consumption Alters the Epigenotype and the Phenotype of Offspring in a Mouse Model
Source: PLoS Genet. 2010 Jan 15;6(1):e1000811. doi: 10.1371/journal.pgen.1000811 (PMC2797299; doi:10.1371/journal.pgen.1000811)
Supplement: Text S1 — Landmark descriptions. (0.03 MB DOC) [file pgen.1000811.s005.doc]

**Dorsal view:**

1-2. Rostral Apex of the nasal bone in dorsal view on left and right sides.

3-4 the maximum width of the nasal bone in dorsal view.

5. The rostral apex of the zygomatic proc. Of left maxilla

6. The rostral apex of the zygomatic proc. Of right maxilla

7. The lateral most point on the left Zygomatic proc. Of frontal

8. The caudal apex of the left nasal.

9. The rostral apex of the frontal (or interfrontal if it exists)

10. The caudal apex of the right nasal.

11. The lateral most point on the right Zygomatic proc. of frontal

12. The rostralmost point on the frontal process of left parietal.

13. The rostralmost point on the medial side of the left parietal.

14. The rostralmost point on the frontal process of right parietal

15-16 Maximum width of the skull measured on the zygomatic process of the squamosals

17-18. Maximum width of the cranium measured on the body of the squamosal

19. The extreme point on the mediocaudal corner of the left parietal.

20. The caudal apex of the interparietal.

**Ventral View**

21. The rostral most point of incisive foramen on the maxilla on the right side

22. The rostral most point of incisive foramen on the maxilla on the left side

23. Medial most point on the maxilla below the premaxilla-maxilla suture right side

24. Medial most point on the maxilla below the premaxilla-maxilla suture right side

25. The caudal most point of the incisive fr. On the right side on the maxilla.

26. The caudal most point of the incisive fr. On the left side on the

27. The mediocaudal of the right maxilla

28. The most caudal aspect of the ultimate molar on the right maxilla

29. The extreme mediocaudal point on the right palatine

30. The most caudal aspect of the ultimate molar on the right maxilla.

31. The median point on the rostral most sphenoid.

32. The median point on the caudal most sphenoid

33. The median caudal most point on the basioccipital.

**Caudal View**

34. The ventral most point on the midline of occipital.

**Anterior View**

35. The most dorsomedial point on the rightside of the nasal opening.
